# Unexplained Rhabdomyolysis and Hepatic Renal Dysfunction: A Case of Bongkrekic Acid Poisoning

**DOI:** 10.7759/cureus.70625

**Published:** 2024-10-01

**Authors:** RongMing Hu, TieGang Fu, SenLin Xia, ShuYing Fei, ZhuXiao Yin

**Affiliations:** 1 Emergency Medicine, Huzhou Central Hospital, Huzhou, CHN; 2 Medicine, Hangzhou Dian Medical Laboratory Co. Ltd., Hangzhou, CHN; 3 Medicine, Zhejiang Wuxing High School, Huzhou, CHN

**Keywords:** bongkrekic acid, burkholderia gladioli, food poisoning, multi-organ dysfunction, prognosis, rhabdomyolysis, tremella

## Abstract

Bongkrekic acid (BA) is a lipotoxin that can cause fatal food poisoning. Severe BA poisoning can rapidly progress from liver and kidney damage to multiple organ failure and is rarely manifested as persistent hypoglycemia and rhabdomyolysis. It has a high mortality rate and poor prognosis. However, clinical data on patients with foodborne BA poisoning are limited. The aim of this study is to summarize the characteristics of patients with BA poisoning, provide reference for early diagnosis and treatment, and improve the survival rate and prognosis of patients with BA poisoning.

## Introduction

Bongkrekic acid (BA) is a lipotoxin produced by the bacterium *Burkholderia gladioli* pathovar cocovenenans (*B. cocovenenans*) [1]. The variant can easily contaminate fermented grain products, *Tremella*, and black fungus, leading to fatal food poisoning [2]. In recent years, it has become a deadly food safety issue, posing a significant challenge to public health [3,4]. Epidemiological investigations have found simultaneous detection of *B. cocovenenans* and BA in coconut and maize products implicated in foodborne outbreaks in Indonesia, China, and Mozambique [5,6]. These outbreaks, primarily associated with coconut and maize products in Indonesia and China but rarely involving *Tremella fuciformis* and *Auricularia auricula*, have resulted in high mortality rates, particularly affecting the liver, kidneys, heart, and brain. The above-mentioned foodborne disease outbreaks are related to the dietary habits of Chinese and Indonesian people and have a certain geographical correlation. Reported mortality rates in China and Indonesia have reached 40% and 60%, respectively, much higher than other common bacterial food poisonings [7]. BA poisoning is extremely dangerous, and patients are generally quickly transferred to the ICU for emergency treatment, creating tremendous pressure on all parties involved [8]. Currently, there is limited clinical data on foodborne BA poisoning. This report describes a patient admitted for diagnosis and treatment of BA poisoning, summarizing the clinical features of BA poisoning to provide a reference for early diagnosis and treatment, thereby improving the survival rate of BA-poisoned patients.

## Case presentation

The patient was a 79-year-old female farmer with a history of hypertension. She received antihypertensive treatment with oral olmesartan medoxomil tablets once a day, and her blood pressure control was satisfactory. The chief complaint was "weakness in limbs, muscle soreness, and black stool for a week," and she came to our hospital for treatment. One week ago, the patient experienced limb weakness, mainly in both lower limbs, showing symmetrical left and right movements. There was also muscle soreness in both lower limbs, and the strength of both upper limbs was still acceptable. There was no limb movement disorder, accompanied by black stool, with a small amount, and bowel movements were relieved one to two times a day. There was no obvious abdominal pain, no bloody stool, no hemoptysis or vomiting blood, no chest pain or tightness, no fear of cold or fever, decreased appetite, and gradually reduced urine output, ultimately leading to oliguria. The patient denied a history of vigorous activity and physical activity before the onset of the disease. Later, the family sent the patient to a local hospital for treatment, including acid suppression, stomach protection, liver protection, and blood dialysis. The patient's condition continued to progress, and the symptoms of limb weakness worsened, accompanied by drowsiness. The family sent the patient to our hospital for further diagnosis and treatment.

Physical examination upon admission revealed body temperature at 36.0℃, blood pressure of 120/52 mmHg (1 mmHg = 0.133 kPa), pulse of 79 times/minute, respiration of 35 times/minute, and fingertip oxygen at 98%. The patient had sleepiness, yellowing of the skin and sclera, slightly low respiratory sounds in both lower lungs, no rales, regular heart rhythm, and no heart murmurs. The abdomen was slightly distended, the abdominal muscles were soft, and there was mild tenderness throughout the abdomen without rebound pain. The bowel sounds were weakened. The urinary catheter was in place, and a small amount of dark brown urine was drained. The lower limbs had blue-purple spots with mild edema, the muscle strength of both lower limbs was level 2, and the muscle strength of both upper limbs was level 5.

**Figure 1:**
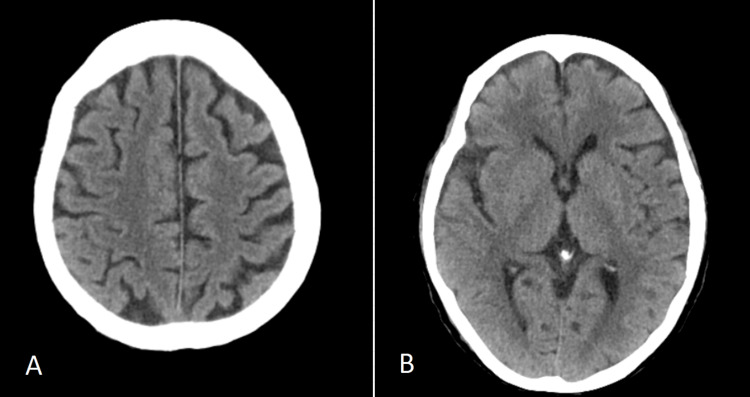
Craniocerebral CT: Mild demyelinating changes in the white matter. Imaging examination results (outpatient department).

**Figure 2:**
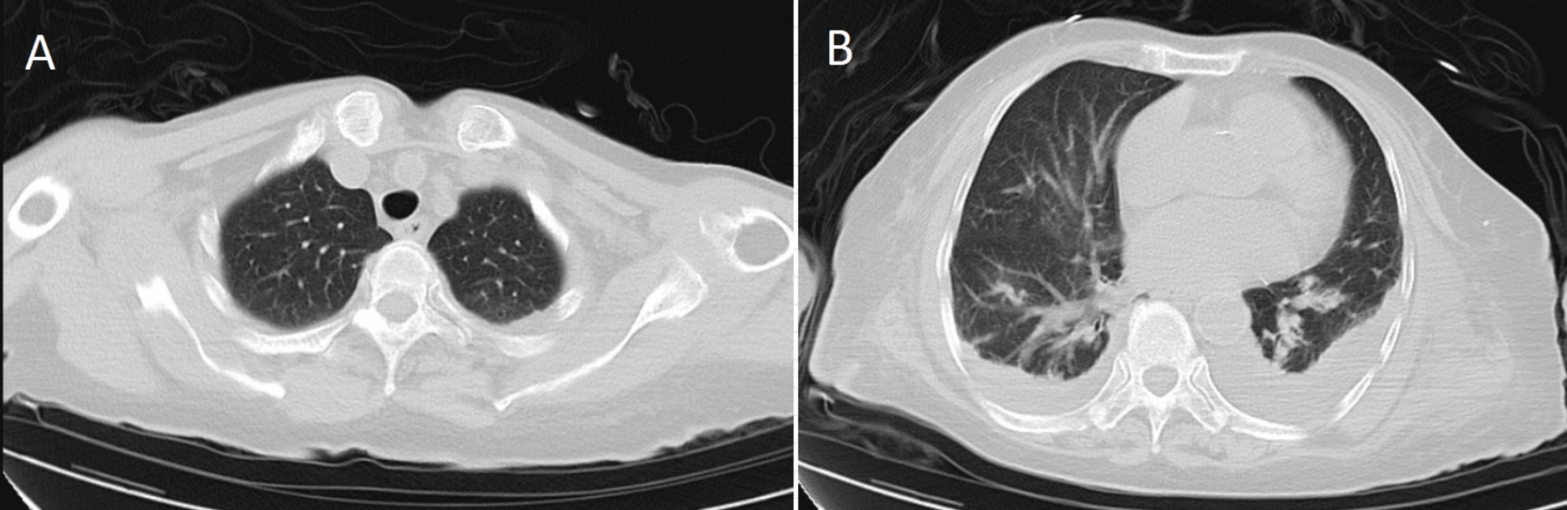
Chest CT: Scattered fibrous exudate lesions in both lungs, with small pleural effusion on both sides and partial lung tissue hypoplasia. Imaging examination results (outpatient department).

**Figure 3:**
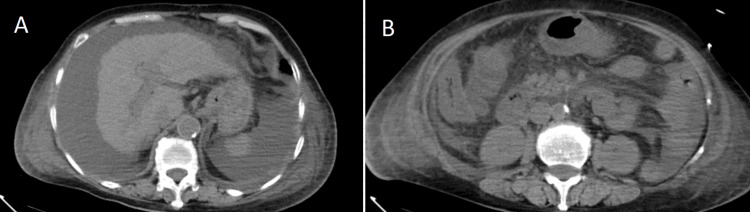
Abdominal CT: (1) Signs of cirrhosis, small amount of fluid accumulation in the abdominal and pelvic cavity, and slightly cloudy peritoneal space. (2) Thickening of the lower esophagus and ascending colon wall. Imaging examination results (outpatient department).

At the time of admission, the bedside electrocardiogram examination was normal. Emergency biochemistry showed blood glucose at 9.72 mmol/L, alanine aminotransferase at 924.2 U/L, aspartate aminotransferase at 1649.2 U/L, total bilirubin at 112.8 umol/L, direct bilirubin at 78.8 umol/L, indirect bilirubin at 34.0 umol/L, albumin at 15.1 g/L, creatinine at 336.1 umol/L, and urea nitrogen at 24.79 mmol/L. Emergency blood routine showed a white blood cell count of 20.0x10 ^ 9/L, high-sensitivity C-reactive protein at 11.5 mg/L, hemoglobin at 78.0 g/L, and platelets at 105.0x10 ^ 9/L. Emergency coagulation parameters revealed a prothrombin time ratio of 2.50, prothrombin time of 28.2 seconds, international normalized ratio (INR) of 2.39, and prothrombin normal control of 11.3 seconds. The creatine kinase level measured by external hospitals was greater than 10000 U/L. The imaging examination results during the outpatient visit are shown in Figures 1-3.

The treatment process and changes in the patient's condition after admission

**Figure 4:**
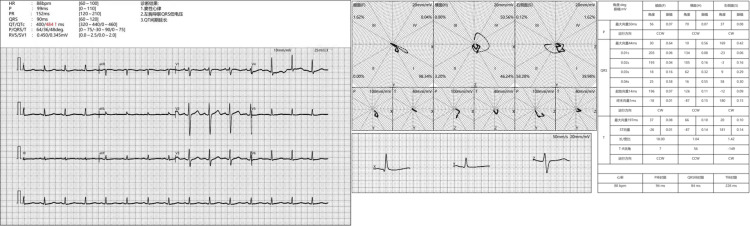
Electrocardiogram: Sinus rhythm, low QRS voltage in the left chest lead, and prolonged QT interval (day one). P wave: The waveform representing atrial contraction in electrocardiogram. PR interval: P wave to QRS complex interval refers to the time interval between atrial and ventricular contractions. QRS complex: It represents the waveform of ventricular contraction. QT: QT interval refers to the time interval during which the ventricle begins to contract and returns to a resting state. QT/QTc: The electrocardiogram QT represents the time required for the entire process of ventricular depolarization and repolarization, while QTc refers to the corrected QT interval. P/QRS/T axis: The P/QRS/T axis usually refers to the P wave (normal range: amplitude ≤ 0.25 mv, width ≤ 0.11 s), QRS complex (normal range: 0.06-0.10s), and T wave (not less than 1/10 of the R wave) on the electrocardiogram examination report. Rv5/Sv1: Rv5 and Sv1 in electrocardiogram refer to the R wave in lead V5 and the S wave in lead V1. CW: Clockwise direction. CCW: Counter clockwise direction. Electrocardiogram examination results (inpatient department).

**Figure 5:**
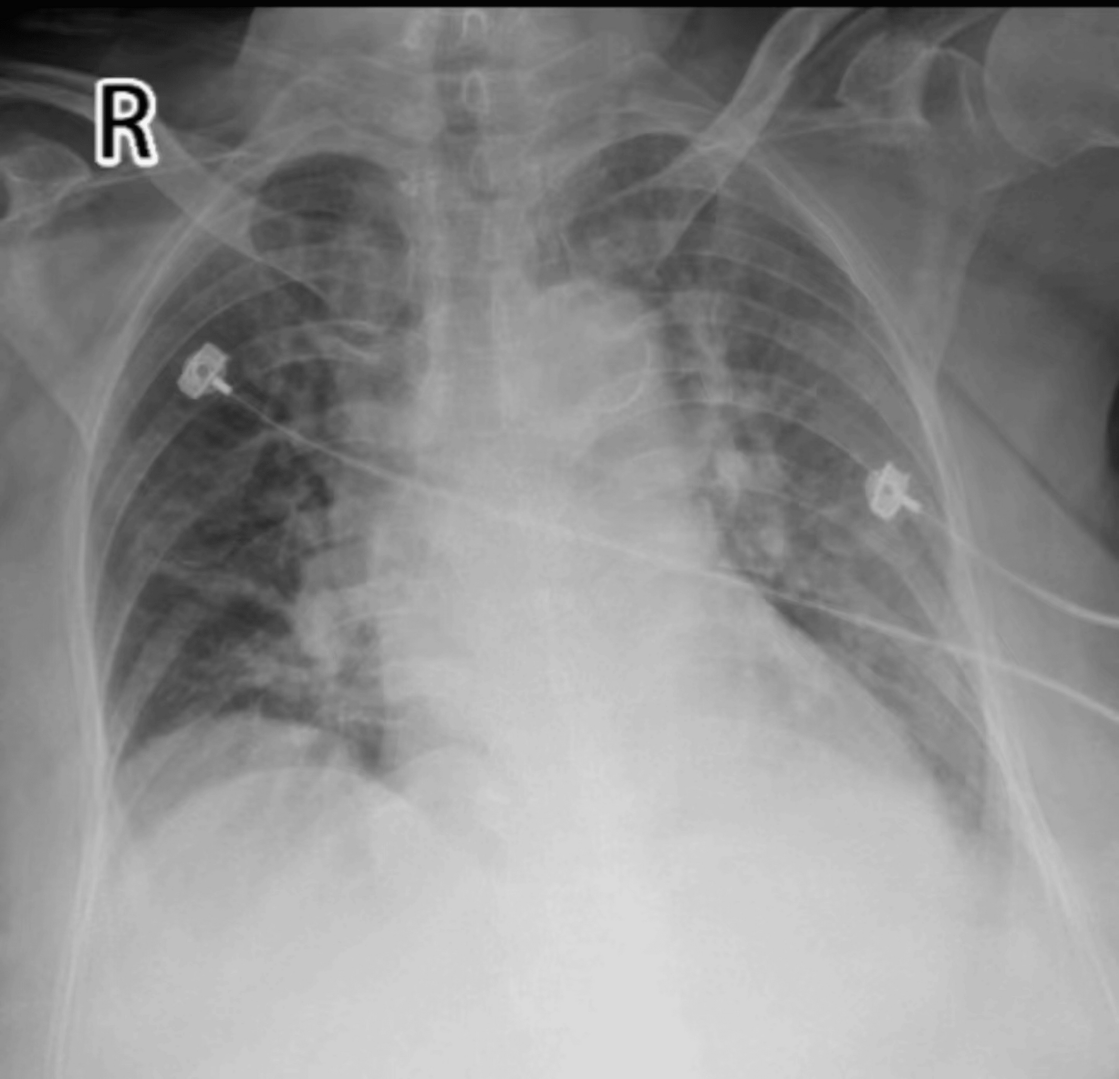
Bedside chest X-ray in the supine position: Scattered exudate lesions in both lungs and left pleural reaction (day one). Imaging examination results (inpatient department).

**Figure 6:**
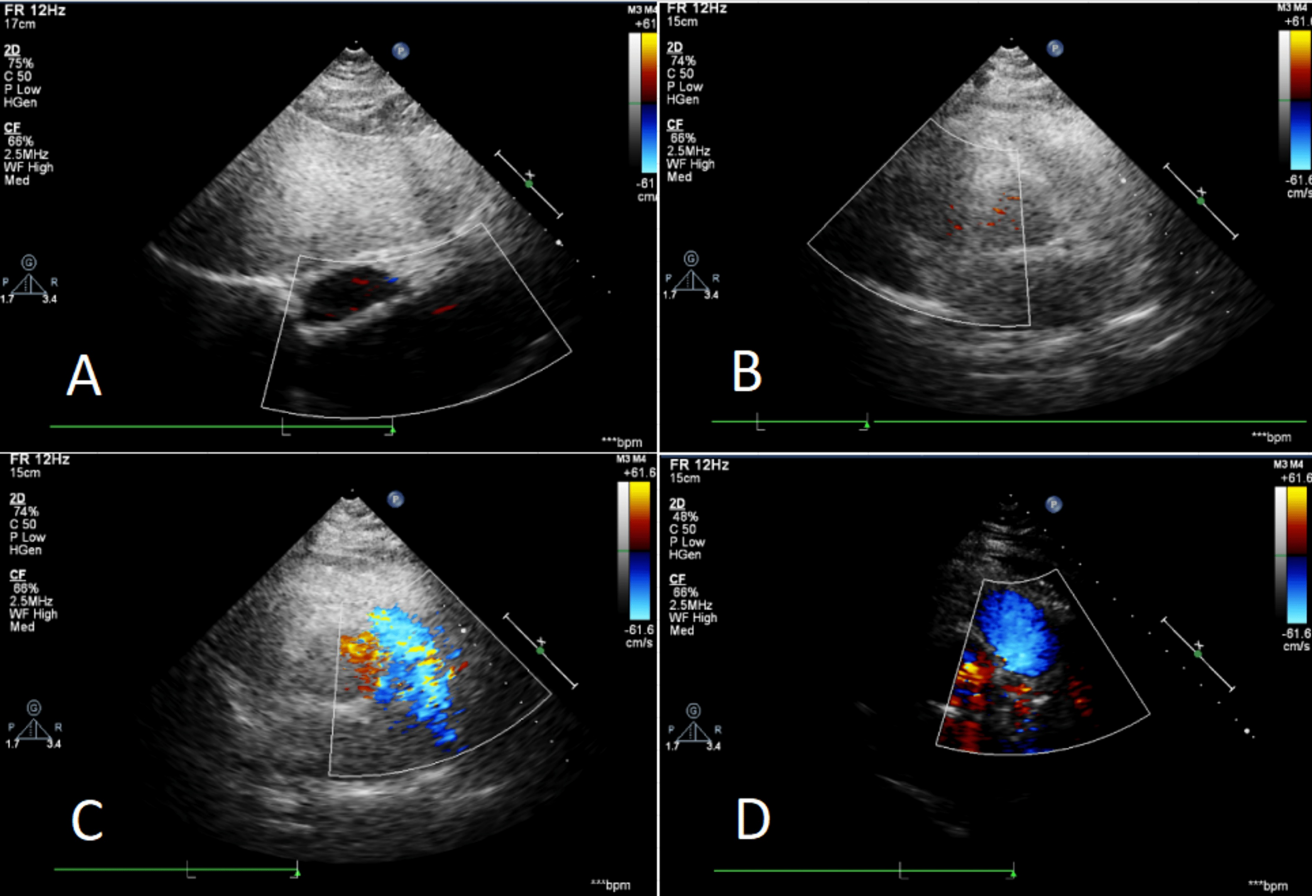
Echocardiography: Enlargement of the left and right atria and aortic sclerosis. Mild regurgitation of the mitral valve, tricuspid valve, and pulmonary valve. Elevated pulmonary artery systolic pressure (mild). Reduced left ventricular diastolic function. AORD = 10 mm, LAD = 33 mm, IVST = 10 mm, PWT = 10 mm, LVDD = 43 mm, LVDS = 25 mm, PAD = 23 mm, FS = 40%, and EF = 72% (day one). AORD: aortic root dimension; LAD: left atrial diameter; IVST: interventricular septum thickness; PWT: left ventricular posterior wall thickness; LVDD: left ventricular end-diastolic diameter; LVDS: left ventricular end-systolic diameter; PAD: pulmonary artery diameter; FS: fractional shortening; EF: ejection fraction. Color Doppler echocardiography examination results (inpatient department).

After admission, complete examinations were conducted, including electrocardiogram, chest X-ray, and ultrasound electrocardiogram, as shown in Figures 4-6.

**Figure 7:**
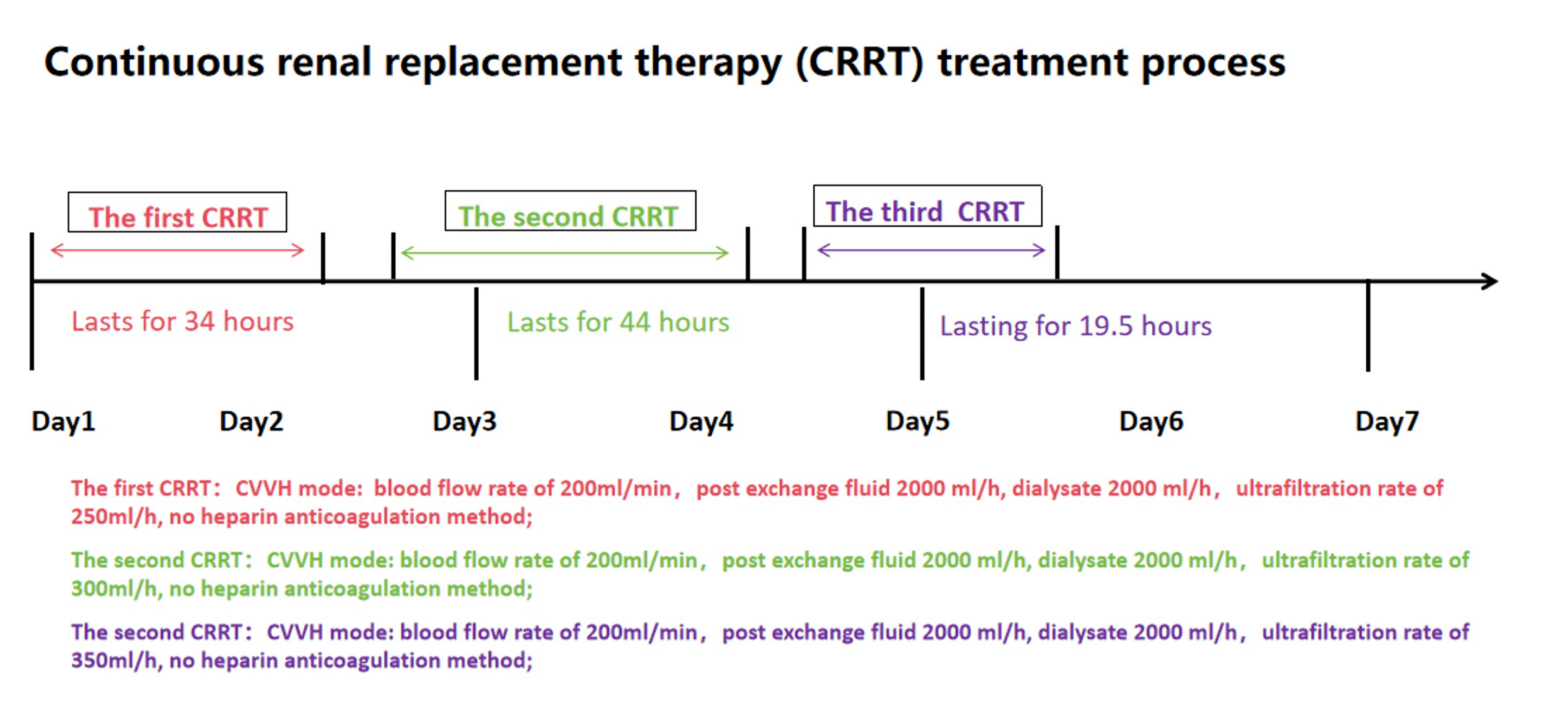
Continuous renal replacement therapy (CRRT) treatment process. CVVH: continuous veno-venous hemofiltration.

The patient's laboratory test results are shown in Tables 1, 2. While assessing the patient's condition after admission, the patient's Sequential Organ Failure Assessment (SOFA) score was 9 points, and elevated C-reactive protein (CRP) levels and white blood cell (WBC) counts were observed, indicating the presence of infection and inflammation. Infection was considered due to the translocation of gut microbiota and the presence of sepsis. Piperacillin sodium and tazobactam sodium injections were given for anti-infection treatment. The levels of aspartate aminotransferase (AST), alanine aminotransferase (ALT), and bilirubin-related indicators (total bilirubin, direct bilirubin, and indirect bilirubin) in the patient were higher than normal levels, while the albumin level was significantly lower than normal levels. At admission, abdominal CT showed signs of liver cirrhosis, so the Child-Pugh modified grading system was applied with a score greater than 10 was noted, indicating severe liver damage and poor liver reserve function. Glutathione injections, adenosine methionine succinate injections, and magnesium isoglycyrrhizinate injections were used to improve liver function and reduce jaundice. Elevated levels of serum creatinine (Sc) and blood urea nitrogen (BUN), combined with oliguria, indicated that renal function was in the decompensated phase of renal failure. Continuous renal replacement therapy (CRRT) was required. The treatment process is shown in Figure 7 and Table 3. Elevated levels of lactate dehydrogenase (LDH) and alpha hydroxybutyrate dehydrogenase (a-HBDH), as well as significantly elevated levels of creatine kinase (CK), ruled out acute liver and heart diseases such as acute myocarditis and acute hepatitis, which may be related to chronic liver cirrhosis. Serious skeletal muscle injury and rhabdomyolysis were considered. The patient was drowsy, although no symptoms such as flapping tremors were observed, and no positive signs of the central nervous system were found during the physical examination. However, the possibility of hepatic encephalopathy was not ruled out. Dynamic monitoring of blood ammonia changes and close monitoring of the patient's mental state changes were done. The patient had intermittent black stool and anemia. The first consideration was upper gastrointestinal bleeding. According to the Glasgow Blatchford score, it is high-risk and indicates a high mortality rate. Close monitoring of hemoglobin changes and black stool symptoms is necessary. Other treatment methods include prohibiting diet, inhibiting gastric acid secretion, supplementing electrolytes such as potassium and sodium, supplementing fluids, infusing albumin, infusing suspended red blood cells, infusing fresh frozen plasma, and providing parenteral nutrition support.

**Table 1:** Laboratory tests during hospitalization.

Laboratory tests	Value								
	Outpatient department	Day 1	Day 2	Day 3	Day 4	Day 5	Day 6	Day 7	Reference range
Hemoglobin (g/L)	78.00	69.00	81.00	78.00	77.00	83.00	76.00	73.00	115.0-150.0
Platelet count (/L)	105.00	115.00	78.00	64.00	55.00	37.00	45.00	47.00	125.0-350.0
White blood cell count (/L)	20.00	18.90	22.40	13.40	12.20	10.70	12.20	13.30	3.5-9.5
C-reactive protein (mg/L)	11.50	10.30	14.40	14.50	10.90	8.40	16.80	30.10	<10.0
Aspartate aminotransferase (U/L)	1649.20	1742.10	1502.20	842.20	378.10	145.80	105.10	72.50	13.0-35.0
Alanine aminotransferase (U/L)	924.20	891.40	883.80	670.50	416.90	247.80	187.50	141.10	7.0-40.0
Total bilirubin (µmol/L)	112.80	114.60	126.90	106.10	84.30	118.30	131.90	170.10	3.4-20.5
Direct bilirubin (µmol/L)	78.80	81.20	84.90	69.40	56.20	70.80	82.00	111.00	0-8.6
Indirect bilirubin (µmol/L)	34.00	33.40	42.00	36.70	28.10	47.50	49.90	59.10	0.1-17.0
Creatinine (µmol/L)	366.10	309.00	139.10	98.90	72.00	83.10	146.10	215.80	41.0-81.0
Blood urea nitrogen (mmol/L)	24.79	22.67	10.48	8.16	5.85	6.54	12.48	21.27	3.1-8.8
Albumin (g/L)	15.10	21.80	25.00	25.00	24.60	23.40	24.20	26.30	40.0-55.0
Creatine kinase (U/L)	Not detected	10601.50	Not detected	Not detected	Not detected	Not detected	Not detected	127.50	26-140
Creatine kinase isoenzymes (U/L)	Not detected	257.60	Not detected	Not detected	Not detected	Not detected	Not detected	36.90	<24.0
Lactate dehydrogenase (U/L)	Not detected	4190.50	Not detected	Not detected	Not detected	Not detected	Not detected	593.60	120-250
Alpha-hydroxybutyrate dehydrogenase (U/L)	Not detected	2239.00	Not detected	Not detected	Not detected	Not detected	Not detected	535.20	72.0-182.0
Procalcitonin (ng/mL)	Not detected	0.97	0.53	0.35	0.27	Not detected	0.60	1.07	<0.065
Prothrombin time (s)	28.20	26.70	23.20	23.30	21.00	22.30	21.70	25.00	9.4-12.5
D-dimer (mg/L)	27.90	23.52	22.12	14.00	18.00	20.61	14.80	14.99	<0.50
Activated partial thromboplastin time (s)	Not detected	Undetectable	40.80	Undetectable	46.60	41.60	56.80	54.60	25.1-36.5
Thrombin time (s)	Not detected	Undetectable	24.80	Undetectable	26.80	Not detected	28.90	24.80	10.3-16.1
International normalized ratio	2.39	2.40	2.08	2.09	1.99	1.88	1.95	2.25	0.83-1.11
Fibrinogen (g/L)	Not detected	0.85	0.93	0.83	0.73	Not detected	0.73	0.86	2.38-4.98
Blood ammonia (umol/L)	71.90	Not detected	63.30	62.90	40.30	Not detected	54.10	Not detected	18-72

**Table 2:** Bedside arterial blood gas analysis results during hospitalization. pH: pH value; PaCO2: arterial blood carbon dioxide partial pressure; PaO2: arterial partial pressure of oxygen; FiO2: fraction of inspired oxygen; Cl-: chloride ion; Na+: sodium ion; K+: potassium ion; Ca2+: calcium ion; HCO3-: bicarbonate ion; Glu: glucose; ABE: actual alkali surplus; SBE: remaining standard alkali; Lac: lactic acid; Hct: hematocrit; mOsm: osmotic pressure.

Test project	Value								
	Outpatient department	Day 1	Day 2	Day 3	Day 4	Day 5	Day 6	Day 7	Reference range
pH	7.400	7.439	7.378	7.400	7.395	7.379	7.379	7.347	7.34-7.45
PaO2 (mmHg)	127.0	122.0	172.0	146.0	174.0	151.0	114.0	168.0	80.0-100.0
PaCO2 (mmHg)	23.9	27.8	32.4	37.0	36.9	41.1	34.2	32.8	35.0-45.0
Cl- (mmol/L)	105.0	105.0	109.0	107.0	108.0	108.0	109.0	108.0	99-110
K+ (mmol/L)	3.7	3.7	4.0	4.0	3.9	3.8	3.8	4.1	3.5-5.5
Na+ (mmol/L)	133.0	135.0	134.0	135.0	135.0	133.0	134.0	133.0	137-147
Ca2+ (mmol/L)	0.81	0.99	1.22	1.22	1.24	1.19	1.20	1.18	1.15-1.29
Glu (mmol/L)	8.9	7.2	8.4	8.4	7.7	7.3	4.4	8.4	3.9-6.2
FiO2 (%)	45.0	45.0	45.0	45.0	45.0	45.0	45.0	45.0	-
HCO3- (mmol/L)	14.5	18.6	18.7	22.4	22.1	23.7	19.7	17.5	21-25
ABE (mmol/L)	-8.9	-4.5	-5.3	-1.6	-1.4	-0.7	-4.3	-7.0	(-3-3)
SBE (mmol/L)	-9.4	-4.9	-5.5	-1.7	-1.6	-0.8	-4.5	-7.2	(-3-3)
Lac (mmol/L)	6.8	4.2	1.5	2.3	1.5	2.3	1.9	2.1	0.5-2.2
Hct (%)	25.50	25.40	27.80	25.90	25.50	27.50	26.30	35.90	35-50
mOsm (mmol/kg)	275.8	271.0	382.0	325.0	387.0	335.0	253.0	372.0	-

**Table 3:** The CRRT treatment parameters. CRRT: continuous renal replacement therapy; CVVH: continuous veno-venous hemofiltration; UFR: ultrafiltration rate; PA: arterial pressure (access pressure); PV: venous pressure (return pressure); TMP: transmembrane pressure.

First CRRT treatment parameters							
Time	Treatment mode	Blood flow velocity (ml/min)	Anticoagulant	UFR (ml/min)	PA (mmHg)	PV (mmHg)	TMP (mmHg)
0:00	CVVH	200	Heparin free	250	-93	73	121
1:00	CVVH	200	Heparin free	250	-88	69	104
2:00	CVVH	200	Heparin free	250	-96	70	133
3:00	CVVH	200	Heparin free	250	-89	74	152
4:00	CVVH	200	Heparin free	250	-90	102	147
5:00	CVVH	200	Heparin free	250	-102	156	139
6:00	CVVH	200	Heparin free	250	-99	155	129
7:00	CVVH	200	Heparin free	250	-104	145	136
8:00	CVVH	200	Heparin free	250	-103	79	110
9:00	CVVH	200	Heparin free	250	-101	80	139
10:00	CVVH	200	Heparin free	250	-105	78	130
11:00	CVVH	200	Heparin free	250	-102	84	131
12:00	CVVH	200	Heparin free	250	-88	93	131
13:00	CVVH	200	Heparin free	250	-88	90	144
14:00	CVVH	200	Heparin free	250	-92	84	149
15:00	CVVH	200	Heparin free	250	-89	84	152
16:00	CVVH	200	Heparin free	250	-92	82	166
17:00	CVVH	200	Heparin free	250	-96	75	183
18:00	CVVH	200	Heparin free	250	-99	81	168
19:00	CVVH	200	Heparin free	250	-97	81	181
20:00	CVVH	200	Heparin free	250	-98	99	188
21:00	CVVH	200	Heparin free	250	-99	91	179
22:00	CVVH	200	Heparin free	250	-97	89	198
23:00	CVVH	200	Heparin free	250	-88	91	182
0:00	CVVH	200	Heparin free	250	-89	92	190
1:00	CVVH	200	Heparin free	250	-90	90	187
2:00	CVVH	200	Heparin free	250	-85	89	188
3:00	CVVH	200	Heparin free	250	-86	92	195
4:00	CVVH	200	Heparin free	250	-83	95	198
5:00	CVVH	200	Heparin free	250	-93	96	200
6:00	CVVH	200	Heparin free	250	-96	98	199
7:00	CVVH	200	Heparin free	250	-99	99	201
8:00	CVVH	200	Heparin free	250	-106	92	197
9:00	CVVH	200	Heparin free	250	-101	81	235
10:00	CVVH	Termination	Termination	Termination	Termination	Termination	Termination
Second CRRT treatment parameters							
Time	Treatment mode	Blood flow velocity (ml/min)	Anticoagulant	UFR (ml/min)	PA (mmHg)	PV (mmHg)	TMP (mmHg)
0:00	CVVH	200	Heparin free	250	-95	86	116
1:00	CVVH	200	Heparin free	250	-90	88	120
2:00	CVVH	200	Heparin free	250	-92	89	123
3:00	CVVH	200	Heparin free	250	-95	90	109
4:00	CVVH	200	Heparin free	250	-99	91	126
5:00	CVVH	200	Heparin free	250	-99	85	141
6:00	CVVH	200	Heparin free	250	-99	86	144
7:00	CVVH	200	Heparin free	300	-93	90	119
8:00	CVVH	200	Heparin free	300	-89	95	125
9:00	CVVH	200	Heparin free	300	-91	90	122
10:00	CVVH	200	Heparin free	300	-92	96	124
11:00	CVVH	200	Heparin free	300	-88	92	122
12:00	CVVH	200	Heparin free	300	-95	102	134
13:00	CVVH	200	Heparin free	300	-92	105	161
14:00	CVVH	200	Heparin free	300	-82	80	169
15:00	CVVH	200	Heparin free	300	-85	80	182
16:00	CVVH	200	Heparin free	300	-88	82	173
17:00	CVVH	200	Heparin free	300	-91	81	142
18:00	CVVH	200	Heparin free	300	-91	84	144
19:00	CVVH	200	Heparin free	300	-95	80	161
20:00	CVVH	200	Heparin free	300	-90	84	179
21:00	CVVH	200	Heparin free	300	-92	86	196
22:00	CVVH	200	Heparin free	300	-92	81	213
23:00	CVVH	200	Heparin free	300	-94	85	206
0:00	CVVH	200	Heparin free	300	-94	86	200
1:00	CVVH	200	Heparin free	300	-93	84	215
2:00	CVVH	200	Heparin free	300	-102	79	163
3:00	CVVH	200	Heparin free	300	-100	75	167
4:00	CVVH	200	Heparin free	300	-104	74	166
5:00	CVVH	200	Heparin free	300	-105	83	221
6:00	CVVH	200	Heparin free	300	-106	85	223
7:00	CVVH	200	Heparin free	300	-108	88	225
8:00	CVVH	200	Heparin free	300	-110	90	214
9:00	CVVH	200	Heparin free	300	-111	98	228
10:00	CVVH	200	Heparin free	300	-113	87	230
11:00	CVVH	200	Heparin free	300	-115	88	231
12:00	CVVH	200	Heparin free	300	-118	90	232
13:00	CVVH	200	Heparin free	300	-102	73	194
14:00	CVVH	200	Heparin free	300	-101	75	201
15:00	CVVH	200	Heparin free	300	-100	84	213
16:00	CVVH	200	Heparin free	300	-100	80	256
17:00	CVVH	200	Heparin free	300	-104	75	258
18:00	CVVH	200	Heparin free	300	-108	79	248
19:00	CVVH	200	Heparin free	300	-102	80	231
20:00	CVVH	Termination	Termination	Termination	Termination	Termination	Termination
Third CRRT treatment parameters							
Time	Treatment mode	Blood flow velocity (ml/min)	Anticoagulant	UFR (ml/min)	PA (mmHg)	PV (mmHg)	TMP (mmHg)
0:00	CVVH	200	Heparin free	350	-81	82	78
1:00	CVVH	200	Heparin free	350	-81	80	102
2:00	CVVH	200	Heparin free	350	-94	87	120
3:00	CVVH	200	Heparin free	350	-95	89	145
4:00	CVVH	200	Heparin free	350	-107	88	140
5:00	CVVH	200	Heparin free	350	-101	89	142
6:00	CVVH	200	Heparin free	350	-101	90	147
7:00	CVVH	200	Heparin free	350	-106	93	183
8:00	CVVH	200	Heparin free	350	-101	89	172
9:00	CVVH	200	Heparin free	350	-105	88	182
10:00	CVVH	200	Heparin free	350	-106	86	191
11:00	CVVH	200	Heparin free	350	-108	89	202
12:00	CVVH	200	Heparin free	350	-124	92	204
13:00	CVVH	200	Heparin free	350	-121	94	234
14:00	CVVH	200	Heparin free	350	-120	92	220
15:00	CVVH	200	Heparin free	350	-136	96	284
16:00	CVVH	200	Heparin free	350	-138	92	267
17:00	CVVH	180	Heparin free	350	-152	48	449
18:00	CVVH	150	Heparin free	350	-141	28	414
19:00	CVVH	150	Heparin free	350	-79	38	443
19:30	CVVH	Termination	Termination	Termination	Termination	Termination	Termination

After further inquiry into the medical history, it was found that the patient had a recent history of continuous consumption of *Tremella fuciformis*. Upon further inquiry, it was found that *Tremella fuciformis* had been soaked overnight multiple times and not consumed in a timely manner or stored in a refrigerator. Therefore, it was suspected that it was food toxin poisoning. After consulting relevant materials and literature, it was considered that the patient was suffering from BA poisoning.

The patient has been taking *Tremella fuciformis* for half a month and has been ill for a week. In the beginning, they were treated with blood dialysis and other treatments at a lower-level hospital. The cause of the illness was unknown and the patient's condition worsened, so they were transferred to our hospital for further diagnosis and treatment. At that time, food poisoning was not considered, so stomach fluid, blood, feces, and other specimens were not collected for toxicological testing. Although our hospital collected blood samples, unfortunately, no toxic substances were detected.

**Figure 8:**
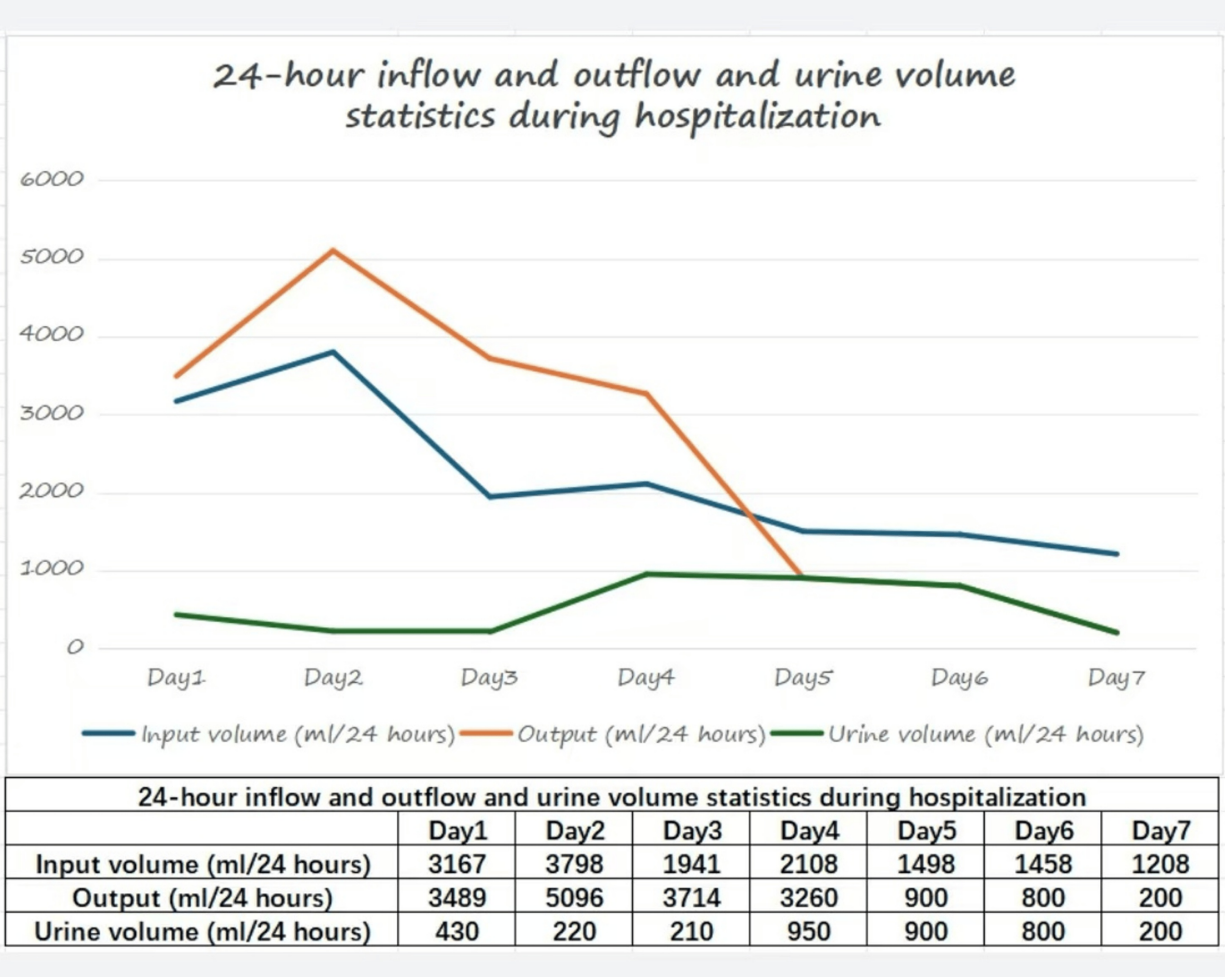
The 24-hour inflow and outflow and urine volume statistics during hospitalization.

On day one of admission, we promptly provided the aforementioned treatment measures, including bedside CRRT. On day two of admission, there was an increasing trend in the patient's inflammatory and infection markers (white blood cell count and CRP), suggesting a possible persistent or worsening inflammatory response. Liver function-related indicators (total bilirubin, direct bilirubin, and indirect bilirubin) all showed varying degrees of elevation, indicating potential liver involvement. Renal function indicators (creatinine and blood urea nitrogen) had significantly decreased, indicating improved renal function. The previous treatment measures still existed. Bedside CRRT was continued for toxin elimination. A consultation with the infectious diseases department was requested, with consideration for plasma exchange if necessary. On day three of admission, reassessment showed improvement in liver enzymes, bilirubin levels, and prothrombin time. Bedside CRRT was continued for toxin clearance, along with the aforementioned treatments. The patient's mental state improved. The patient was allowed to consume a semi-liquid diet. On the fourth day of admission, liver enzyme, bilirubin levels, and prothrombin time further improved. The 24-hour urine output was 950 ml. Bedside CRRT was continued to clear toxins and improve kidney function while receiving other supportive treatments. On day five of admission, liver and kidney function continued to improve, urine output increased, and the 24-hour urine volume was 900 ml. The 24-hour urine flow changes after admission are shown in Figure 8. The CRRT was temporarily stopped, and other supportive treatments continued. On day six of admission, there were signs of deterioration in liver function again, and kidney function had not yet reached its optimal state. The 24-hour urine output was 800 ml and diuretic treatment was initiated. CRRT was restarted. Other supportive treatments were still ongoing. On day seven of admission, liver function continued to worsen, with increased bilirubin levels and prolonged prothrombin time, indicating severe liver dysfunction. Blood exchange transfusion for bilirubin removal and artificial liver support therapy were planned.

The patient suddenly experienced hemoptysis and acute respiratory distress. Emergency tracheal intubation was performed at the bedside, and mechanical ventilation was connected to a ventilator for rescue. The patient's respiratory function had improved. At the same time, supportive treatment was provided to improve coagulation. However, considering the poor prognosis of the patient, the patient's family gave up subsequent treatment and requested to be discharged and return home. After fully evaluating the patient's condition and communicating with their family, the treatment team agreed to discharge the patient and terminate the treatment. Unfortunately, the patient passed away during the follow-up call.

## Discussion

BA is a crucial lethal toxin, characterized as an odorless, tasteless, and heat-resistant substance. Contaminated food substances can retain their normal appearance, odor, and taste, making BA detection challenging during food preparation and consumption. This compound is not expected to volatilize or hydrolyze in the environment; however, exposure to ultraviolet light or sunlight can degrade its chemical structure, reducing or eliminating its toxicity [9,10]. It is noteworthy that cooking contaminated food with BA does not render it safe for consumption, as the toxin itself is heat-stable despite bacterial destruction [1-3,11].

In the hot and humid summer, fermented corn flour products, rice noodles, river noodles, and other wet rice noodles, as well as soaked *Tremella*, agaric, and other foods are easily contaminated by bacteria to produce BA. The food that causes BA has a common feature in the production process: it requires long-term fermentation or soaking, especially in summer and autumn when the temperature and humidity are appropriate. Bacteria are prone to contamination and proliferation, leading to frequent poisoning incidents.

Previous literature has shown that food poisoning incidents involving BA mainly occur in specific regions of Indonesia, China, and some parts of Africa, mainly related to the consumption of locally produced fermented foods [12,13]. Due to a lack of vigilance and attention, diagnosis and treatment have been delayed, affecting the prognosis of patients and increasing the burden on local medical and health resources.

The incubation period of BA poisoning is short, with symptoms possibly appearing as early as 30 minutes post-exposure, or as late as 12 hours, and in rare cases, even one to two days later, with a maximum duration of three days [7]. The target organs of BA action in humans are major parenchymal organs such as the liver, brain, and kidneys [6,8,14]. The main symptoms and signs during the incubation period include damage to the nervous, digestive, and urinary systems. Mild poisoning manifests as dizziness, generalized weakness, upper abdominal discomfort, nausea, vomiting, and mild diarrhea, while severe poisoning presents with coffee ground-like vomiting, altered consciousness, restlessness, jaundice, hepatomegaly, subcutaneous bleeding, hematemesis, hematuria, oliguria, convulsions, seizures, and in the most severe cases, shock, and death. Severe patients often present with hepatic coma, central nervous system paralysis, and death due to respiratory failure. Besides, animal experiments have demonstrated that BA can induce neutrophil extracellular traps via p38, ERK, PAD4, and P2X1-mediated signaling or inhibit the electrical activity of pancreatic beta cells in mice [15,16]. BA binds to the adenine nucleotide translocator in mitochondria, altering its conformation, inhibiting the opening of the mitochondrial permeability transition pore, and interfering with the exchange of adenosine diphosphate and adenosine triphosphate (ATP) on the inner mitochondrial membrane, leading to reduced or absent ATP generation, thereby disrupting the physiological mechanism of ATP dependence in mitochondria and ultimately causing cell death. Clinical manifestations and symptoms of human poisoning are similar to those of other mitochondrial toxins but differ in severity and temporal progression [17]. It has been observed that there is a dose-response relationship between the amount of BA-contaminated food ingested and the severity of the disease [11,18].

In this case, the main symptoms of the patient are limb weakness and muscle pain, manifested as early unexplained rhabdomyolysis and liver and kidney dysfunction, accompanied by atypical gastrointestinal symptoms (black stool) and neurological symptoms (drowsiness). These non-specific symptoms make early diagnosis difficult. Due to the unclear initial cause, the treatment lacked purpose. After admission, the patient's medical history, especially their diet, was thoroughly investigated. The final diagnosis was considered to be BA poisoning caused by an unclean diet of *Tremella fuciformis*, and specific toxicological damage was brought to attention. Unfortunately, due to the prolonged duration of the patient's condition and previous treatment at a lower-level hospital, we were unable to obtain direct evidence of toxin poisoning. Currently, there are no specific antidotes for BA poisoning or standardized treatment guidelines for BA toxicity [10]. In addition, there is still a lack of research on the toxicokinetics of BA, and the pathological and physiological changes caused by toxins are not fully understood.

This case is relatively rare in clinical practice. The early main symptoms are unexplained rhabdomyolysis and liver and kidney dysfunction, followed by rapid onset and progression to multiple organ dysfunction, especially liver function, kidney function, and coagulation dysfunction. Although both lower-level hospitals and our hospital have conducted hemodialysis treatment (CRRT), and there has been a transient improvement in monitoring indicators such as liver function, kidney function, and coagulation function during the course of the disease, early plasma exchange was not performed in a timely manner, resulting in a poor prognosis for the patient. The main reason for treatment failure is the initial lack of a clear diagnosis of the disease, which prevented the early initiation of hemodialysis treatment and also affected the effectiveness of plasma exchange.

The author attempts to summarize the characteristics of BA poisoning to provide early diagnostic and treatment references, thereby improving the survival rate of poisoned patients. Common features of BA poisoning include the following: (1) renal dysfunction, with elevated creatinine and blood urea nitrogen, progressive decrease in urine output, oliguria, anuria, or even renal failure; (2) hepatic dysfunction, with peak transaminase levels reached within three days, possible bile enzyme separation, and hepatocyte necrosis; (3) systemic inflammatory response, characterized by a significant increase in leukocytes, normal C-reactive protein, and a slight increase in procalcitonin; (4) coagulation dysfunction, with prolonged prothrombin time, normal activated partial thromboplastin time, elevated D-dimer, and decreased platelets; (5) metabolic acidosis, with increased lactate levels; (6) generalized weakness in patients, with normal levels of myoglobin and creatine kinase in the early stage (first eight hours), normal electrolytes and renal function, and mild elevation of creatine kinase; (7) abdominal CT imaging may reveal diffuse liver lesions; (8) the severity of poisoning is dose- and time-dependent, with onset of multiple organ dysfunction within three days, or even death. Special attention should be paid to the fact that some cases have also reported unexplained persistent hypoglycemia, and the mechanism is not yet clear, which may be related to the toxin causing liver cell degeneration and affecting liver metabolism [14-16].

Special attention should be paid to the fact that some cases have also reported unexplained persistent hypoglycemia [19], and the mechanism of occurrence is not fully understood. Studies have shown that BA can inhibit enzymatic activity and function through non-covalent binding of thiol groups [20]. After consuming a certain amount of BA, it activates ATP-dependent potassium channels to inhibit pancreatic beta cell function, causing transient hyperglycemia and subsequently turning into hypoglycemia [15,21,22]. Under low glucose conditions, BA induces necrosis of glycogen storage cells such as muscles and liver through glycolysis, leading to digestive and neurological discomfort symptoms such as nausea, vomiting, mild diarrhea, and dizziness in the early stages of poisoning [15,18,21,22].

Therefore, the key to the treatment of BA poisoning is rapid diagnosis. For patients with a short history of ingestion and a high-risk food history of ingesting BA toxins, medical institutions with conditions should promptly perform gastric lavage on patients, while paying attention to preserving gastric lavage fluid. The gastric lavage fluid, vomitus, residual food, and the patient's whole blood should be sent to specialized institutions for quantitative detection of BA content [23,24]. Rapid latex agglutination tests can be used to differentiate BA poisoning [25-29]. However, in most medical institutions lacking diagnostic capabilities, sporadic cases may be missed or misdiagnosed.

At present, due to the obvious regional distribution characteristics of BA, researchers in most countries have paid little attention to it. Scholars outside China mainly use the specific binding of BA to the adenosine transfer site of adenine nucleotide translocator (ANT) to study mitochondrial function and its molecular mechanism. However, the mechanism of toxicity is not yet clear, and the research on specific detoxification drugs based on specific sites is also not yet perfect. For the above-mentioned problems of BA and BA poisoning, researchers from all over the world, including China, need to work together to solve them.

In addition to strengthening early diagnosis and timely treatment of BA poisoning, it is also necessary to improve the prevention of BA poisoning. This requires strict implementation of food safety regulations and standards by countries around the world, improvement of food safety risk monitoring and assessment systems, and promotion of upgrading of food safety risk communication models. Based on the existing popularization of food safety education, it is necessary to understand the public's cognitive level and adopt different communication models to improve certain unhealthy production, processing, and dietary habits of the public according to their cognitive level.

In high-risk areas, preventive measures should be taken to avoid contact with *B. cocovenenans* and BA, and safer fermentation processes should be adopted. In addition, training on BA poisoning and diagnostic testing in primary healthcare institutions should be strengthened to enhance timely diagnosis and emergency response capabilities. When patients experience unexplained liver and kidney dysfunction combined with rhabdomyolysis, BA poisoning should be considered early based on medical history, symptoms, signs, and laboratory test results, and plasma exchange therapy should be performed promptly.

## Conclusions

In summary, BA poisoning progresses rapidly and has a high mortality rate, often leading to multiple organ dysfunction, including liver and kidney damage. Although there is a lack of specific detoxifying agents for the BA toxin produced by *Burkholderia gladioli *(*B. cocovenenans*), early diagnosis based on the clinical history and timely adjustment of treatment strategies, especially early plasma exchange, and necessary hemodialysis, can help reduce mortality and improve patient prognosis.
